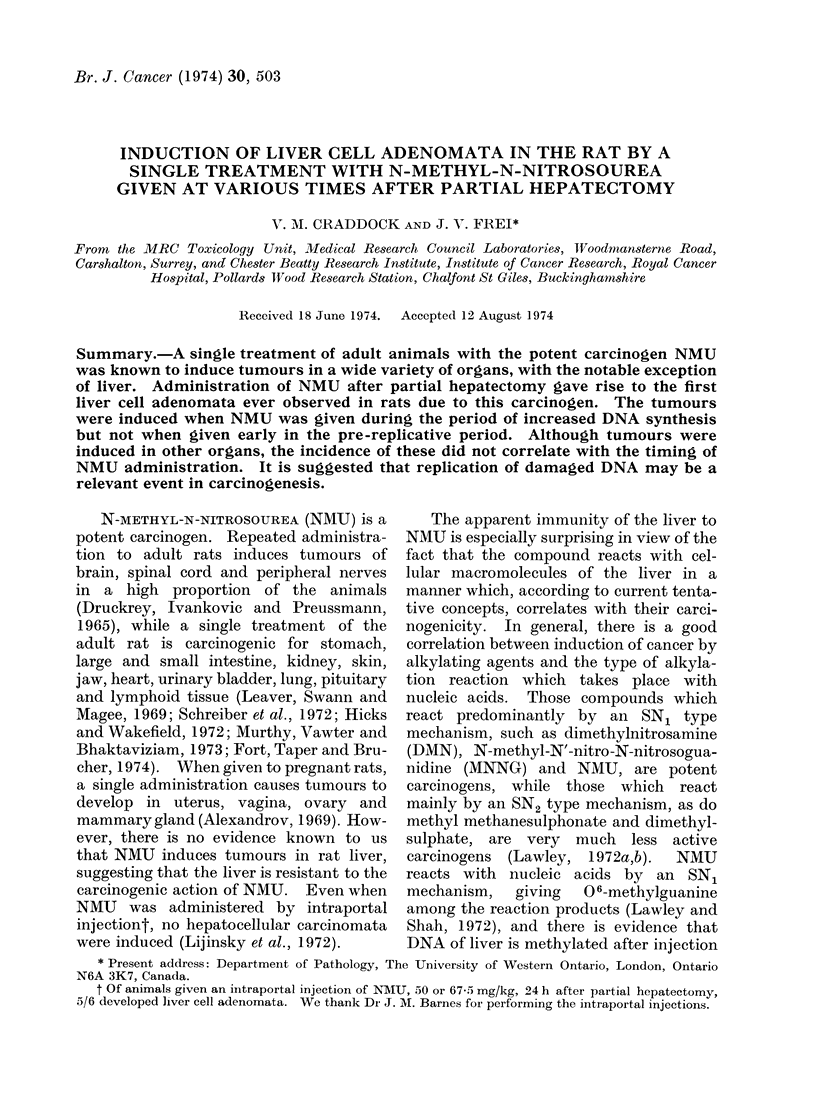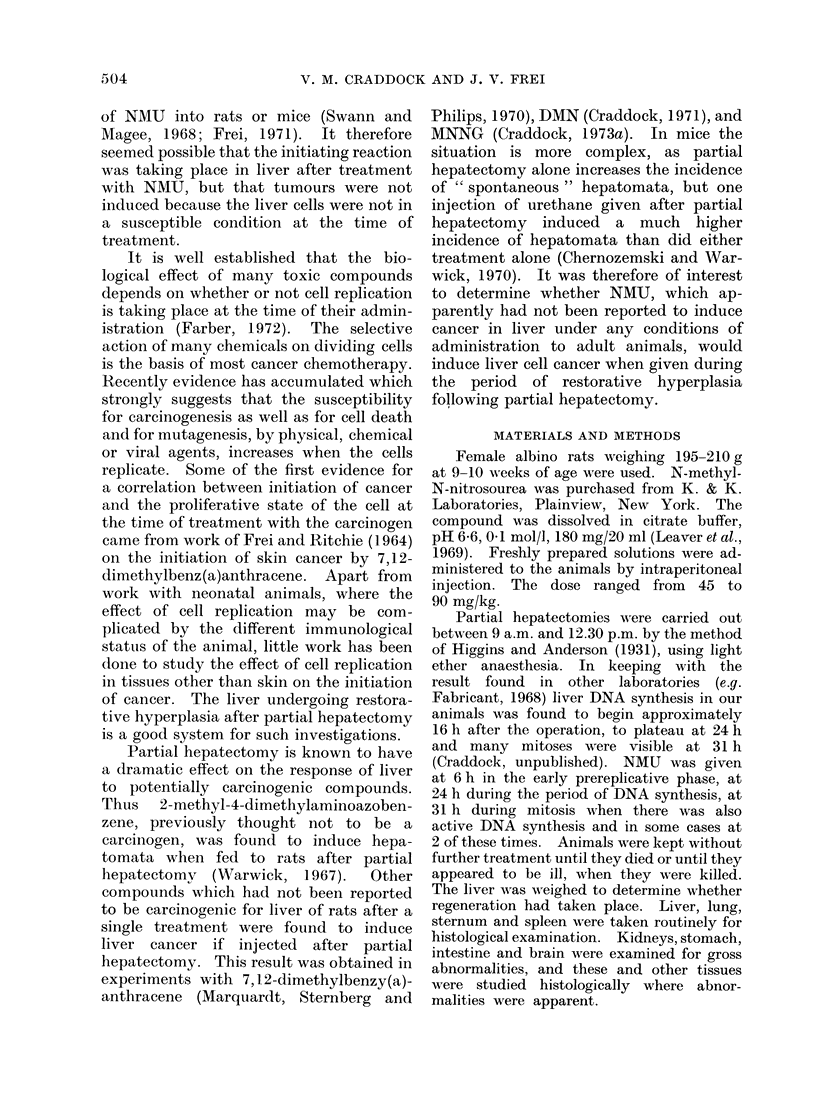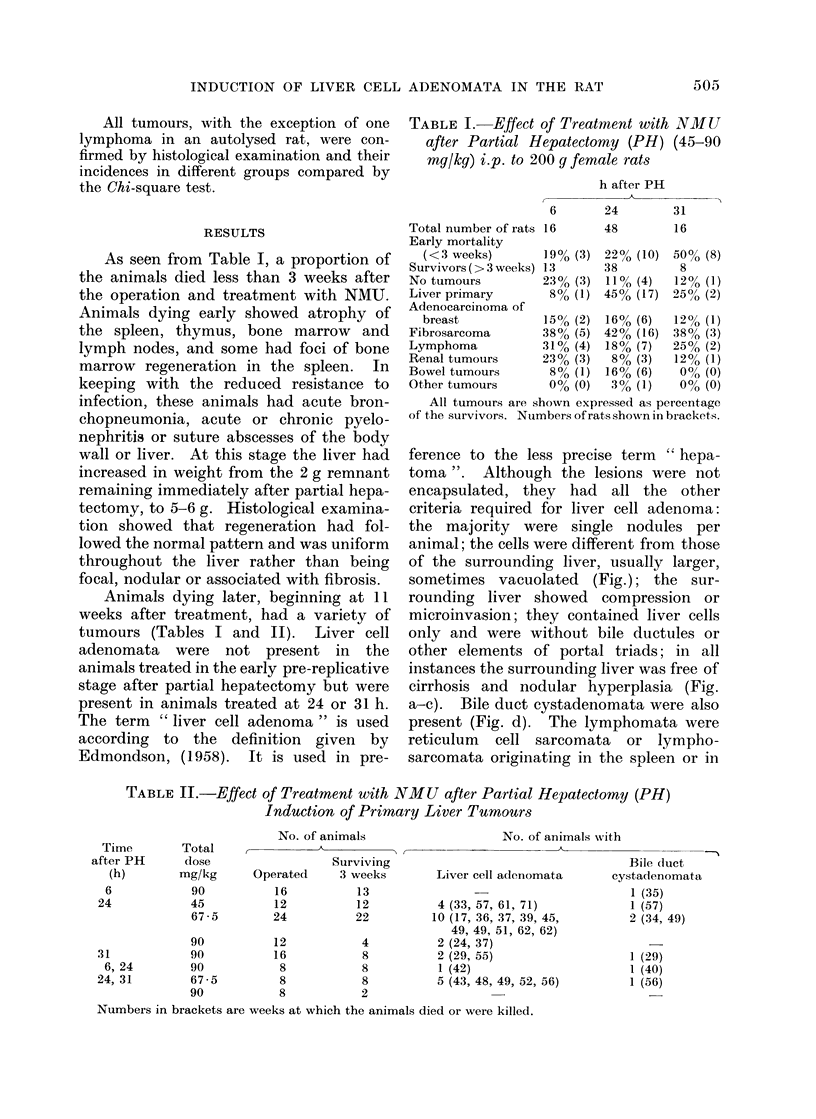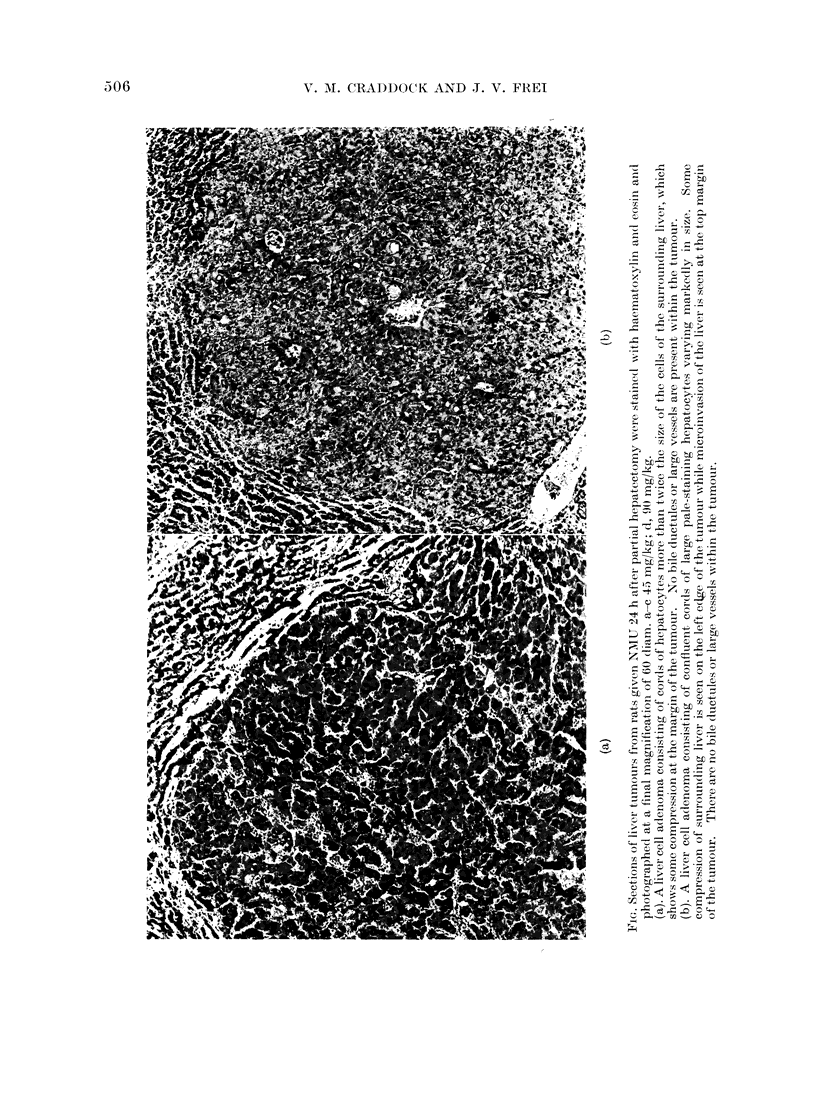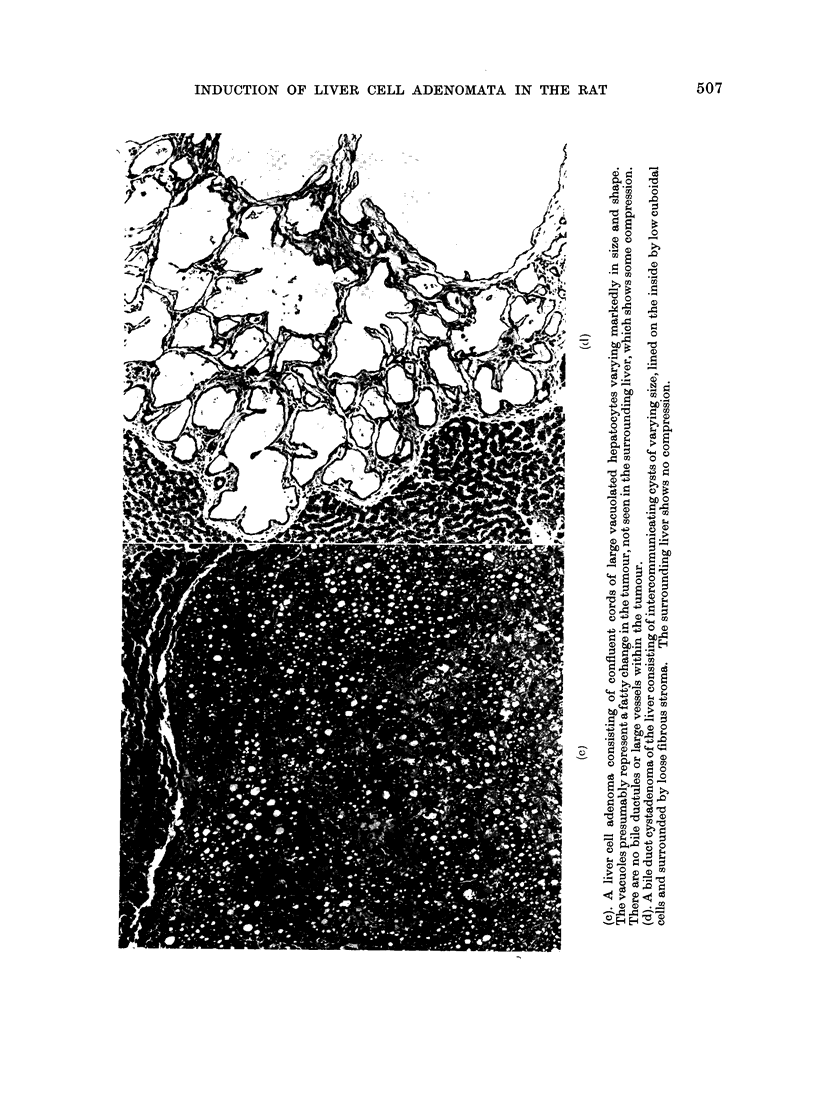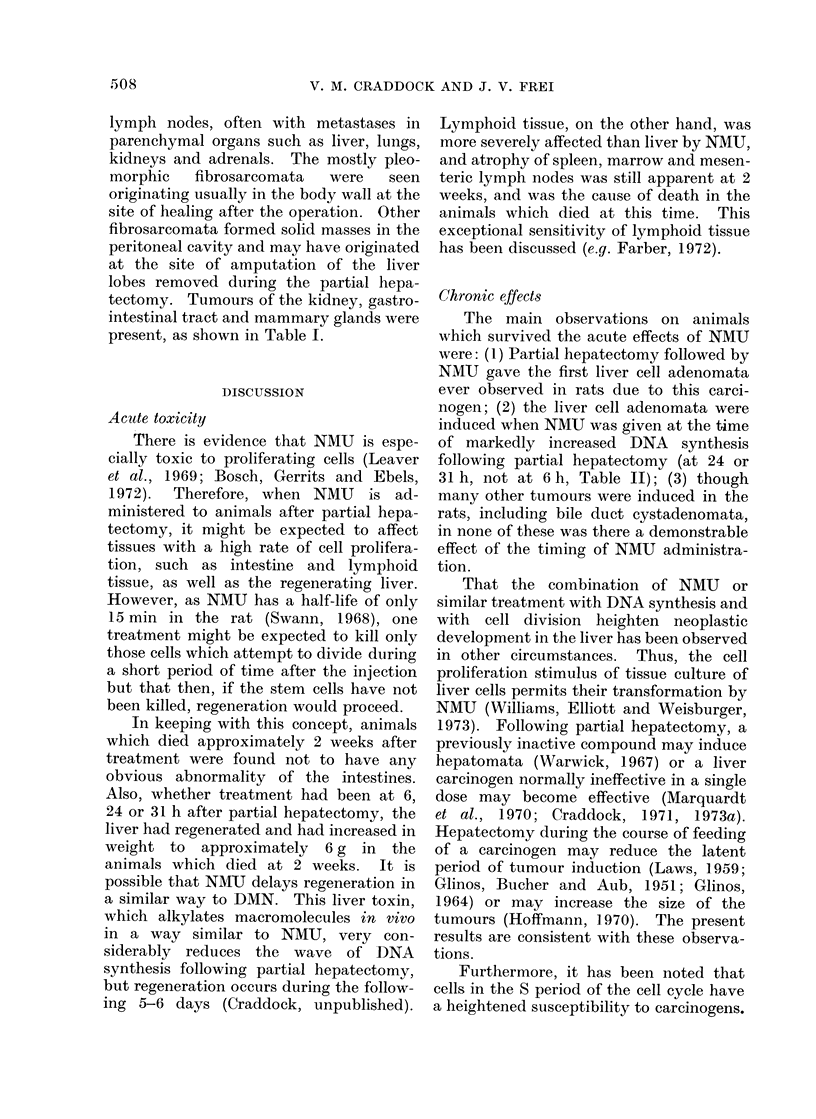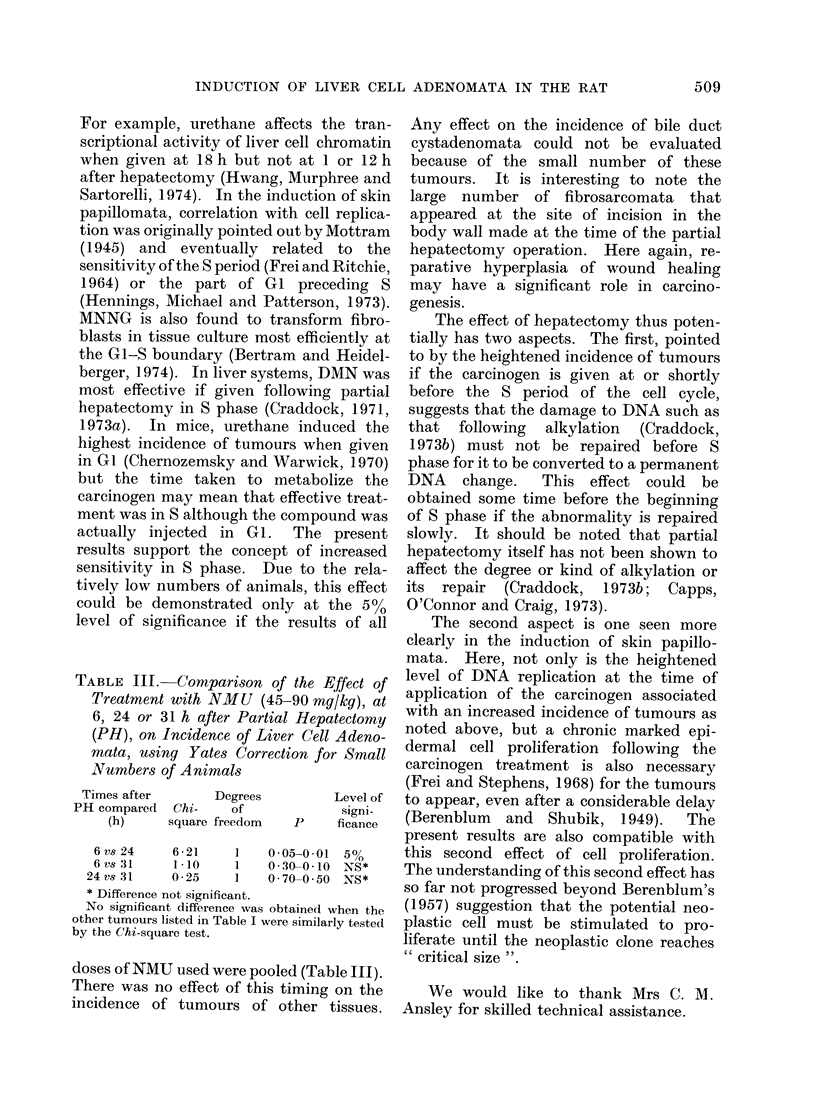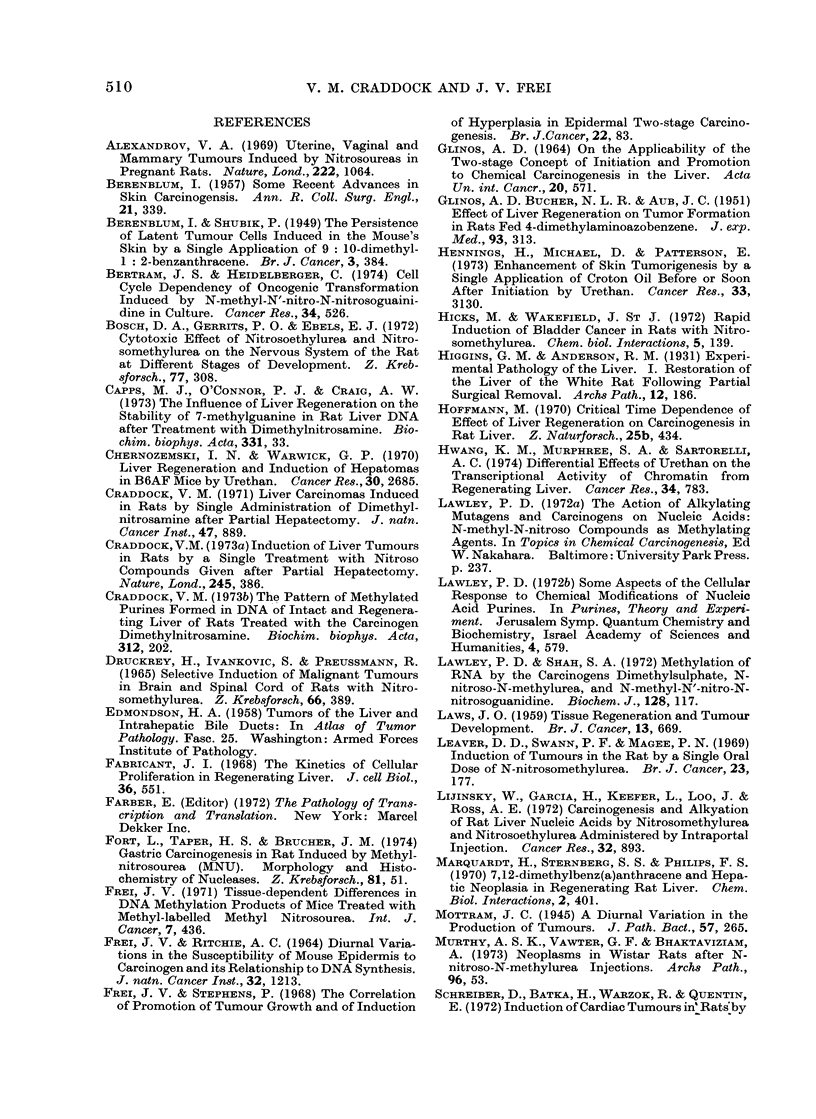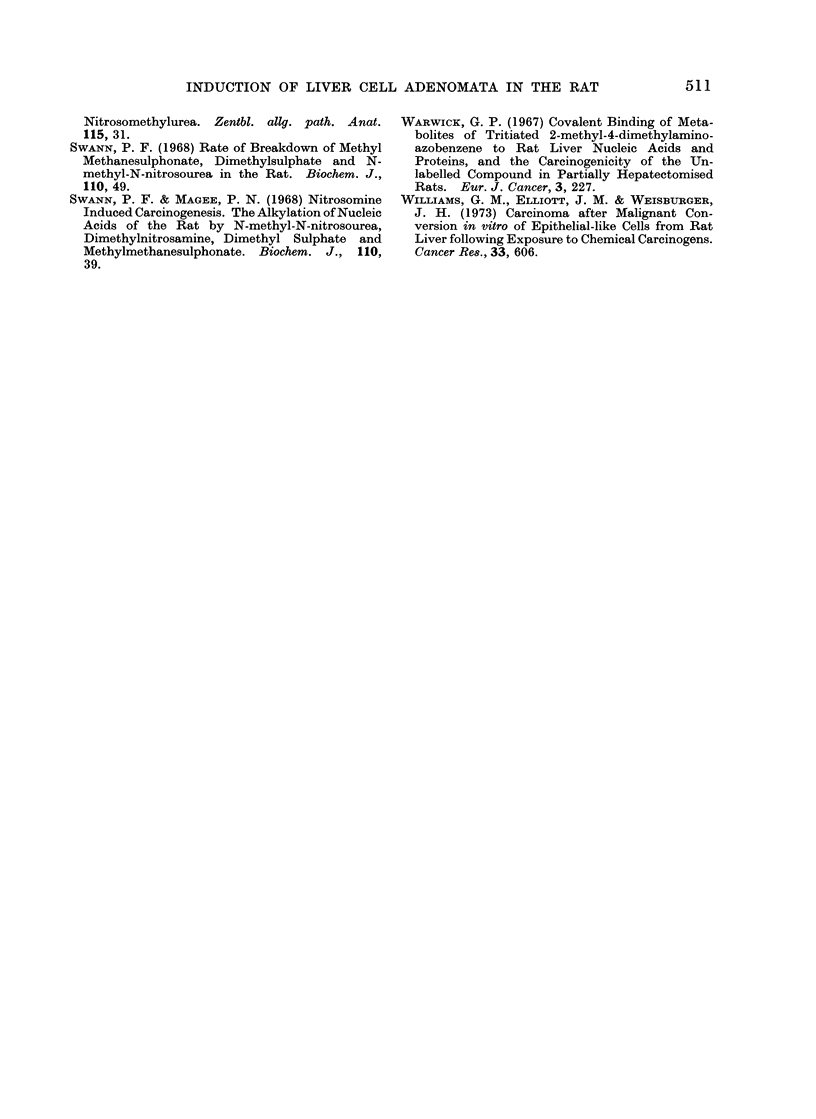# Induction of Liver Cell Adenomata in the Rat by a Single Treatment with N-Methyl-N-Nitrosourea given at Various Times after Partial Hepatectomy

**DOI:** 10.1038/bjc.1974.229

**Published:** 1974-12

**Authors:** V. M. Craddock, J. V. Frei

## Abstract

**Images:**


Br. J. Cancer (1974) 30, 503

### INDUCTION OF LIVER CELL ADENOMATA IN THE RAT BY A SINGLE TREATMENT WITH N-METHYL-N-NITROSOUREA GIVEN AT VARIOUS TIMES AFTER PARTIAL HEPATECTOMY

V. M. CRADDOCK AND J. V. FREI*

*From the MRC Toxicology Unit, Medical Research Council Laboratories, Woodmansterne Road, Carshalton, Surrey, and Chester Beatty Research Institute, Institute of Cancer Research, Royal Cancer Hospital, Pollards Wood Research Station, Chalfont St Giles, Buckinghamshire*

Received 18 June 1974. Accepted 12 August 1974

**Summary.—A single treatment of adult animals with the potent carcinogen NMU was known to induce tumours in a wide variety of organs, with the notable exception of liver. Administration of NMU after partial hepatectomy gave rise to the first liver cell adenomata ever observed in rats due to this carcinogen. The tumours were induced when NMU was given during the period of increased DNA synthesis but not when given early in the pre-replicative period. Although tumours were induced in other organs, the incidence of these did not correlate with the timing of NMU administration. It is suggested that replication of damaged DNA may be a relevant event in carcinogenesis.**

N-METHYL-N-NITROSOUREA (NMU) is a potent carcinogen. Repeated administration to adult rats induces tumours of brain, spinal cord and peripheral nerves in a high proportion of the animals (Druckrey, Ivankovic and Preussmann, 1965), while a single treatment of the adult rat is carcinogenic for stomach, large and small intestine, kidney, skin, jaw, heart, urinary bladder, lung, pituitary and lymphoid tissue (Leaver, Swann and Magee, 1969; Schreiber *et al.*, 1972; Hicks and Wakefield, 1972; Murthy, Vawter and Bhaktaviziam, 1973; Fort, Taper and Brucher, 1974). When given to pregnant rats, a single administration causes tumours to develop in uterus, vagina, ovary and mammary gland (Alexandrov, 1969). However, there is no evidence known to us that NMU induces tumours in rat liver, suggesting that the liver is resistant to the carcinogenic action of NMU. Even when NMU was administered by intraportal injection†, no hepatocellular carcinomata were induced (Lijinsky *et al.*, 1972).

The apparent immunity of the liver to NMU is especially surprising in view of the fact that the compound reacts with cellular macromolecules of the liver in a manner which, according to current tentative concepts, correlates with their carcinogenicity. In general, there is a good correlation between induction of cancer by alkylating agents and the type of alkylation reaction which takes place with nucleic acids. Those compounds which react predominantly by an $SN_1$ type mechanism, such as dimethylnitrosamine (DMN), N-methyl-N′-nitro-N-nitrosoguanidine (MNNG) and NMU, are potent carcinogens, while those which react mainly by an $SN_2$ type mechanism, as do methyl methanesulphonate and dimethylsulphate, are very much less active carcinogens (Lawley, 1972*a*,*b*). NMU reacts with nucleic acids by an $SN_1$ mechanism, giving $O^6$-methylguanine among the reaction products (Lawley and Shah, 1972), and there is evidence that DNA of liver is methylated after injection

* Present address: Department of Pathology, The University of Western Ontario, London, Ontario N6A 3K7, Canada.

† Of animals given an intraportal injection of NMU, 50 or 67·5 mg/kg, 24 h after partial hepatectomy, 5/6 developed liver cell adenomata. We thank Dr J. M. Barnes for performing the intraportal injections.

of NMU into rats or mice (Swann and Magee, 1968; Frei, 1971). It therefore seemed possible that the initiating reaction was taking place in liver after treatment with NMU, but that tumours were not induced because the liver cells were not in a susceptible condition at the time of treatment.

It is well established that the biological effect of many toxic compounds depends on whether or not cell replication is taking place at the time of their administration (Farber, 1972). The selective action of many chemicals on dividing cells is the basis of most cancer chemotherapy. Recently evidence has accumulated which strongly suggests that the susceptibility for carcinogenesis as well as for cell death and for mutagenesis, by physical, chemical or viral agents, increases when the cells replicate. Some of the first evidence for a correlation between initiation of cancer and the proliferative state of the cell at the time of treatment with the carcinogen came from work of Frei and Ritchie (1964) on the initiation of skin cancer by 7,12-dimethylbenz(a)anthracene. Apart from work with neonatal animals, where the effect of cell replication may be complicated by the different immunological status of the animal, little work has been done to study the effect of cell replication in tissues other than skin on the initiation of cancer. The liver undergoing restorative hyperplasia after partial hepatectomy is a good system for such investigations.

Partial hepatectomy is known to have a dramatic effect on the response of liver to potentially carcinogenic compounds. Thus 2-methyl-4-dimethylaminoazobenzene, previously thought not to be a carcinogen, was found to induce hepatomata when fed to rats after partial hepatectomy (Warwick, 1967). Other compounds which had not been reported to be carcinogenic for liver of rats after a single treatment were found to induce liver cancer if injected after partial hepatectomy. This result was obtained in experiments with 7,12-dimethylbenzy(a)-anthracene (Marquardt, Sternberg and Philips, 1970), DMN (Craddock, 1971), and MNNG (Craddock, 1973*a*). In mice the situation is more complex, as partial hepatectomy alone increases the incidence of " spontaneous " hepatomata, but one injection of urethane given after partial hepatectomy induced a much higher incidence of hepatomata than did either treatment alone (Chernozemski and Warwick, 1970). It was therefore of interest to determine whether NMU, which apparently had not been reported to induce cancer in liver under any conditions of administration to adult animals, would induce liver cell cancer when given during the period of restorative hyperplasia following partial hepatectomy.

### MATERIALS AND METHODS

Female albino rats weighing 195–210 g at 9–10 weeks of age were used. N-methyl-N-nitrosourea was purchased from K. & K. Laboratories, Plainview, New York. The compound was dissolved in citrate buffer, pH 6·6, 0·1 mol/l, 180 mg/20 ml (Leaver *et al.*, 1969). Freshly prepared solutions were administered to the animals by intraperitoneal injection. The dose ranged from 45 to 90 mg/kg.

Partial hepatectomies were carried out between 9 a.m. and 12.30 p.m. by the method of Higgins and Anderson (1931), using light ether anaesthesia. In keeping with the result found in other laboratories (*e.g.* Fabricant, 1968) liver DNA synthesis in our animals was found to begin approximately 16 h after the operation, to plateau at 24 h and many mitoses were visible at 31 h (Craddock, unpublished). NMU was given at 6 h in the early prereplicative phase, at 24 h during the period of DNA synthesis, at 31 h during mitosis when there was also active DNA synthesis and in some cases at 2 of these times. Animals were kept without further treatment until they died or until they appeared to be ill, when they were killed. The liver was weighed to determine whether regeneration had taken place. Liver, lung, sternum and spleen were taken routinely for histological examination. Kidneys, stomach, intestine and brain were examined for gross abnormalities, and these and other tissues were studied histologically where abnormalities were apparent.

All tumours, with the exception of one lymphoma in an autolysed rat, were confirmed by histological examination and their incidences in different groups compared by the *Chi*-square test.

### RESULTS

As seen from Table I, a proportion of the animals died less than 3 weeks after the operation and treatment with NMU. Animals dying early showed atrophy of the spleen, thymus, bone marrow and lymph nodes, and some had foci of bone marrow regeneration in the spleen. In keeping with the reduced resistance to infection, these animals had acute bronchopneumonia, acute or chronic pyelonephritis or suture abscesses of the body wall or liver. At this stage the liver had increased in weight from the 2 g remnant remaining immediately after partial hepatectomy, to 5–6 g. Histological examination showed that regeneration had followed the normal pattern and was uniform throughout the liver rather than being focal, nodular or associated with fibrosis.

Animals dying later, beginning at 11 weeks after treatment, had a variety of tumours (Tables I and II). Liver cell adenomata were not present in the animals treated in the early pre-replicative stage after partial hepatectomy but were present in animals treated at 24 or 31 h. The term " liver cell adenoma " is used according to the definition given by Edmondson, (1958). It is used in preference to the less precise term " hepatoma ". Although the lesions were not encapsulated, they had all the other criteria required for liver cell adenoma: the majority were single nodules per animal; the cells were different from those of the surrounding liver, usually larger, sometimes vacuolated (Fig.); the surrounding liver showed compression or microinvasion; they contained liver cells only and were without bile ductules or other elements of portal triads; in all instances the surrounding liver was free of cirrhosis and nodular hyperplasia (Fig. a–c). Bile duct cystadenomata were also present (Fig. d). The lymphomata were reticulum cell sarcomata or lymphosarcomata originating in the spleen or in

TABLE I.—*Effect of Treatment with NMU after Partial Hepatectomy (PH) (45–90 mg/kg) i.p. to 200 g female rats*

| | h after PH | | |
|---|---|---|---|
| | 6 | 24 | 31 |
| Total number of rats | 16 | 48 | 16 |
| Early mortality (<3 weeks) | 19% (3) | 22% (10) | 50% (8) |
| Survivors (>3 weeks) | 13 | 38 | 8 |
| No tumours | 23% (3) | 11% (4) | 12% (1) |
| Liver primary | 8% (1) | 45% (17) | 25% (2) |
| Adenocarcinoma of breast | 15% (2) | 16% (6) | 12% (1) |
| Fibrosarcoma | 38% (5) | 42% (16) | 38% (3) |
| Lymphoma | 31% (4) | 18% (7) | 25% (2) |
| Renal tumours | 23% (3) | 8% (3) | 12% (1) |
| Bowel tumours | 8% (1) | 16% (6) | 0% (0) |
| Other tumours | 0% (0) | 3% (1) | 0% (0) |

All tumours are shown expressed as percentage of the survivors. Numbers of rats shown in brackets.

TABLE II.—*Effect of Treatment with NMU after Partial Hepatectomy (PH) Induction of Primary Liver Tumours*

| Time after PH (h) | Total dose mg/kg | No. of animals: Operated | Surviving 3 weeks | No. of animals with: Liver cell adenomata | Bile duct cystadenomata |
|---|---|---|---|---|---|
| 6 | 90 | 16 | 13 | — | 1 (35) |
| 24 | 45 | 12 | 12 | 4 (33, 57, 61, 71) | 1 (57) |
| | 67·5 | 24 | 22 | 10 (17, 36, 37, 39, 45, 49, 49, 51, 62, 62) | 2 (34, 49) |
| | 90 | 12 | 4 | 2 (24, 37) | — |
| 31 | 90 | 16 | 8 | 2 (29, 55) | 1 (29) |
| 6, 24 | 90 | 8 | 8 | 1 (42) | 1 (40) |
| 24, 31 | 67·5 | 8 | 8 | 5 (43, 48, 49, 52, 56) | 1 (56) |
| | 90 | 8 | 2 | — | — |

Numbers in brackets are weeks at which the animals died or were killed.

lymph nodes, often with metastases in parenchymal organs such as liver, lungs, kidneys and adrenals. The mostly pleomorphic fibrosarcomata were seen originating usually in the body wall at the site of healing after the operation. Other fibrosarcomata formed solid masses in the peritoneal cavity and may have originated at the site of amputation of the liver lobes removed during the partial hepatectomy. Tumours of the kidney, gastrointestinal tract and mammary glands were present, as shown in Table I.

### DISCUSSION

### *Acute toxicity*

There is evidence that NMU is especially toxic to proliferating cells (Leaver *et al.*, 1969; Bosch, Gerrits and Ebels, 1972). Therefore, when NMU is administered to animals after partial hepatectomy, it might be expected to affect tissues with a high rate of cell proliferation, such as intestine and lymphoid tissue, as well as the regenerating liver. However, as NMU has a half-life of only 15 min in the rat (Swann, 1968), one treatment might be expected to kill only those cells which attempt to divide during a short period of time after the injection but that then, if the stem cells have not been killed, regeneration would proceed.

In keeping with this concept, animals which died approximately 2 weeks after treatment were found not to have any obvious abnormality of the intestines. Also, whether treatment had been at 6, 24 or 31 h after partial hepatectomy, the liver had regenerated and had increased in weight to approximately 6 g in the animals which died at 2 weeks. It is possible that NMU delays regeneration in a similar way to DMN. This liver toxin, which alkylates macromolecules *in vivo* in a way similar to NMU, very considerably reduces the wave of DNA synthesis following partial hepatectomy, but regeneration occurs during the following 5–6 days (Craddock, unpublished). Lymphoid tissue, on the other hand, was more severely affected than liver by NMU, and atrophy of spleen, marrow and mesenteric lymph nodes was still apparent at 2 weeks, and was the cause of death in the animals which died at this time. This exceptional sensitivity of lymphoid tissue has been discussed (*e.g.* Farber, 1972).

### *Chronic effects*

The main observations on animals which survived the acute effects of NMU were: (1) Partial hepatectomy followed by NMU gave the first liver cell adenomata ever observed in rats due to this carcinogen; (2) the liver cell adenomata were induced when NMU was given at the time of markedly increased DNA synthesis following partial hepatectomy (at 24 or 31 h, not at 6 h, Table II); (3) though many other tumours were induced in the rats, including bile duct cystadenomata, in none of these was there a demonstrable effect of the timing of NMU administration.

That the combination of NMU or similar treatment with DNA synthesis and with cell division heighten neoplastic development in the liver has been observed in other circumstances. Thus, the cell proliferation stimulus of tissue culture of liver cells permits their transformation by NMU (Williams, Elliott and Weisburger, 1973). Following partial hepatectomy, a previously inactive compound may induce hepatomata (Warwick, 1967) or a liver carcinogen normally ineffective in a single dose may become effective (Marquardt *et al.*, 1970; Craddock, 1971, 1973*a*). Hepatectomy during the course of feeding of a carcinogen may reduce the latent period of tumour induction (Laws, 1959; Glinos, Bucher and Aub, 1951; Glinos, 1964) or may increase the size of the tumours (Hoffmann, 1970). The present results are consistent with these observations.

Furthermore, it has been noted that cells in the S period of the cell cycle have a heightened susceptibility to carcinogens.

For example, urethane affects the transcriptional activity of liver cell chromatin when given at 18 h but not at 1 or 12 h after hepatectomy (Hwang, Murphree and Sartorelli, 1974). In the induction of skin papillomata, correlation with cell replication was originally pointed out by Mottram (1945) and eventually related to the sensitivity of the S period (Frei and Ritchie, 1964) or the part of G1 preceding S (Hennings, Michael and Patterson, 1973). MNNG is also found to transform fibroblasts in tissue culture most efficiently at the G1–S boundary (Bertram and Heidelberger, 1974). In liver systems, DMN was most effective if given following partial hepatectomy in S phase (Craddock, 1971, 1973*a*). In mice, urethane induced the highest incidence of tumours when given in G1 (Chernozemsky and Warwick, 1970) but the time taken to metabolize the carcinogen may mean that effective treatment was in S although the compound was actually injected in G1. The present results support the concept of increased sensitivity in S phase. Due to the relatively low numbers of animals, this effect could be demonstrated only at the 5% level of significance if the results of all doses of NMU used were pooled (Table III). There was no effect of this timing on the incidence of tumours of other tissues.

TABLE III.—*Comparison of the Effect of Treatment with NMU* (45–90 *mg/kg*), *at* 6, 24 *or* 31 *h after Partial Hepatectomy* (*PH*), *on Incidence of Liver Cell Adenomata, using Yates Correction for Small Numbers of Animals*

| Times after PH compared (h) | *Chi*-square | Degrees of freedom | *P* | Level of significance |
|---|---|---|---|---|
| 6 *vs* 24 | 6·21 | 1 | 0·05–0·01 | 5% |
| 6 *vs* 31 | 1·10 | 1 | 0·30–0·10 | NS* |
| 24 *vs* 31 | 0·25 | 1 | 0·70–0·50 | NS* |

* Difference not significant.

No significant difference was obtained when the other tumours listed in Table I were similarly tested by the *Chi*-square test.

Any effect on the incidence of bile duct cystadenomata could not be evaluated because of the small number of these tumours. It is interesting to note the large number of fibrosarcomata that appeared at the site of incision in the body wall made at the time of the partial hepatectomy operation. Here again, reparative hyperplasia of wound healing may have a significant role in carcinogenesis.

The effect of hepatectomy thus potentially has two aspects. The first, pointed to by the heightened incidence of tumours if the carcinogen is given at or shortly before the S period of the cell cycle, suggests that the damage to DNA such as that following alkylation (Craddock, 1973*b*) must not be repaired before S phase for it to be converted to a permanent DNA change. This effect could be obtained some time before the beginning of S phase if the abnormality is repaired slowly. It should be noted that partial hepatectomy itself has not been shown to affect the degree or kind of alkylation or its repair (Craddock, 1973*b*; Capps, O'Connor and Craig, 1973).

The second aspect is one seen more clearly in the induction of skin papillomata. Here, not only is the heightened level of DNA replication at the time of application of the carcinogen associated with an increased incidence of tumours as noted above, but a chronic marked epidermal cell proliferation following the carcinogen treatment is also necessary (Frei and Stephens, 1968) for the tumours to appear, even after a considerable delay (Berenblum and Shubik, 1949). The present results are also compatible with this second effect of cell proliferation. The understanding of this second effect has so far not progressed beyond Berenblum's (1957) suggestion that the potential neoplastic cell must be stimulated to proliferate until the neoplastic clone reaches " critical size ".

We would like to thank Mrs C. M. Ansley for skilled technical assistance.

### REFERENCES

ALEXANDROV, V. A. (1969) Uterine, Vaginal and Mammary Tumours Induced by Nitrosoureas in Pregnant Rats. *Nature, Lond.*, **222**, 1064.

BERENBLUM, I. (1957) Some Recent Advances in Skin Carcinogensis. *Ann. R. Coll. Surg. Engl.*, **21**, 339.

BERENBLUM, I. & SHUBIK, P. (1949) The Persistence of Latent Tumour Cells Induced in the Mouse's Skin by a Single Application of 9 : 10-dimethyl-1 : 2-benzanthracene. *Br. J. Cancer*, **3**, 384.

BERTRAM, J. S. & HEIDELBERGER, C. (1974) Cell Cycle Dependency of Oncogenic Transformation Induced by N-methyl-N′-nitro-N-nitrosoguainidine in Culture. *Cancer Res.*, **34**, 526.

BOSCH, D. A., GERRITS, P. O. & EBELS, E. J. (1972) Cytotoxic Effect of Nitrosoethylurea and Nitrosomethylurea on the Nervous System of the Rat at Different Stages of Development. *Z. Krebsforsch.*, **77**, 308.

CAPPS, M. J., O'CONNOR, P. J. & CRAIG, A. W. (1973) The Influence of Liver Regeneration on the Stability of 7-methylguanine in Rat Liver DNA after Treatment with Dimethylnitrosamine. *Biochim. biophys. Acta*, **331**, 33.

CHERNOZEMSKI, I. N. & WARWICK, G. P. (1970) Liver Regeneration and Induction of Hepatomas in B6AF Mice by Urethan. *Cancer Res.*, **30**, 2685.

CRADDOCK, V. M. (1971) Liver Carcinomas Induced in Rats by Single Administration of Dimethylnitrosamine after Partial Hepatectomy. *J. natn. Cancer Inst.*, **47**, 889.

CRADDOCK, V.M. (1973*a*) Induction of Liver Tumours in Rats by a Single Treatment with Nitroso Compounds Given after Partial Hepatectomy. *Nature, Lond.*, **245**, 386.

CRADDOCK, V. M. (1973*b*) The Pattern of Methylated Purines Formed in DNA of Intact and Regenerating Liver of Rats Treated with the Carcinogen Dimethylnitrosamine. *Biochim. biophys. Acta*, **312**, 202.

DRUCKREY, H., IVANKOVIC, S. & PREUSSMANN, R. (1965) Selective Induction of Malignant Tumours in Brain and Spinal Cord of Rats with Nitrosomethylurea. *Z. Krebsforsch*, **66**, 389.

EDMONDSON, H. A. (1958) Tumors of the Liver and Intrahepatic Bile Ducts: In *Atlas of Tumor Pathology*. Fasc. 25. Washington: Armed Forces Institute of Pathology.

FABRICANT, J. I. (1968) The Kinetics of Cellular Proliferation in Regenerating Liver. *J. cell Biol.*, **36**, 551.

FARBER, E. (Editor) (1972) *The Pathology of Transcription and Translation.* New York: Marcel Dekker Inc.

FORT, L., TAPER, H. S. & BRUCHER, J. M. (1974) Gastric Carcinogenesis in Rat Induced by Methylnitrosourea (MNU). Morphology and Histochemistry of Nucleases. *Z. Krebsforsch.*, **81**, 51.

FREI, J. V. (1971) Tissue-dependent Differences in DNA Methylation Products of Mice Treated with Methyl-labelled Methyl Nitrosourea. *Int. J. Cancer*, **7**, 436.

FREI, J. V. & RITCHIE, A. C. (1964) Diurnal Variations in the Susceptibility of Mouse Epidermis to Carcinogen and its Relationship to DNA Synthesis. *J. natn. Cancer Inst.*, **32**, 1213.

FREI, J. V. & STEPHENS, P. (1968) The Correlation of Promotion of Tumour Growth and of Induction of Hyperplasia in Epidermal Two-stage Carcinogenesis. *Br. J.Cancer*, **22**, 83.

GLINOS, A. D. (1964) On the Applicability of the Two-stage Concept of Initiation and Promotion to Chemical Carcinogenesis in the Liver. *Acta Un. int. Cancr.*, **20**, 571.

GLINOS, A. D. BUCHER, N. L. R. & AUB, J. C. (1951) Effect of Liver Regeneration on Tumor Formation in Rats Fed 4-dimethylaminoazobenzene. *J. exp. Med.*, **93**, 313.

HENNINGS, H., MICHAEL, D. & PATTERSON, E. (1973) Enhancement of Skin Tumorigenesis by a Single Application of Croton Oil Before or Soon After Initiation by Urethan. *Cancer Res.*, **33**, 3130.

HICKS, M. & WAKEFIELD, J. ST J. (1972) Rapid Induction of Bladder Cancer in Rats with Nitrosomethylurea. *Chem. biol. Interactions*, **5**, 139.

HIGGINS, G. M. & ANDERSON, R. M. (1931) Experimental Pathology of the Liver. I. Restoration of the Liver of the White Rat Following Partial Surgical Removal. *Archs Path.*, **12**, 186.

HOFFMANN, M. (1970) Critical Time Dependence of Effect of Liver Regeneration on Carcinogenesis in Rat Liver. *Z. Naturforsch.*, **25b**, 434.

HWANG, K. M., MURPHREE, S. A. & SARTORELLI, A. C. (1974) Differential Effects of Urethan on the Transcriptional Activity of Chromatin from Regenerating Liver. *Cancer Res.*, **34**, 783.

LAWLEY, P. D. (1972*a*) The Action of Alkylating Mutagens and Carcinogens on Nucleic Acids: N-methyl-N-nitroso Compounds as Methylating Agents. In *Topics in Chemical Carcinogenesis*, Ed W. Nakahara. Baltimore: University Park Press. p. 237.

LAWLEY, P. D. (1972*b*) Some Aspects of the Cellular Response to Chemical Modifications of Nucleic Acid Purines. In *Purines, Theory and Experiment.* Jerusalem Symp. Quantum Chemistry and Biochemistry, Israel Academy of Sciences and Humanities, **4**, 579.

LAWLEY, P. D. & SHAH, S. A. (1972) Methylation of RNA by the Carcinogens Dimethylsulphate, N-nitroso-N-methylurea, and N-methyl-N′-nitro-N-nitrosoguanidine. *Biochem. J.*, **128**, 117.

LAWS, J. O. (1959) Tissue Regeneration and Tumour Development. *Br. J. Cancer*, **13**, 669.

LEAVER, D. D., SWANN, P. F. & MAGEE, P. N. (1969) Induction of Tumours in the Rat by a Single Oral Dose of N-nitrosomethylurea. *Br. J. Cancer*, **23**, 177.

LIJINSKY, W., GARCIA, H., KEEFER, L., LOO, J. & ROSS, A. E. (1972) Carcinogenesis and Alkyation of Rat Liver Nucleic Acids by Nitrosomethylurea and Nitrosoethylurea Administered by Intraportal Injection. *Cancer Res.*, **32**, 893.

MARQUARDT, H., STERNBERG, S. S. & PHILIPS, F. S. (1970) 7,12-dimethylbenz(a)anthracene and Hepatic Neoplasia in Regenerating Rat Liver. *Chem. Biol. Interactions*, **2**, 401.

MOTTRAM, J. C. (1945) A Diurnal Variation in the Production of Tumours. *J. Path. Bact.*, **57**, 265.

MURTHY, A. S. K., VAWTER, G. F. & BHAKTAVIZIAM, A. (1973) Neoplasms in Wistar Rats after N-nitroso-N-methylurea Injections. *Archs Path.*, **96**, 53.

SCHREIBER, D., BATKA, H., WARZOK, R. & QUENTIN, E. (1972) Induction of Cardiac Tumours in Rats by

Nitrosomethylurea. *Zentbl. allg. path. Anat.* **115**, 31.

SWANN, P. F. (1968) Rate of Breakdown of Methyl Methanesulphonate, Dimethylsulphate and N-methyl-N-nitrosourea in the Rat. *Biochem. J.*, **110**, 49.

SWANN, P. F. & MAGEE, P. N. (1968) Nitrosomine Induced Carcinogenesis. The Alkylation of Nucleic Acids of the Rat by N-methyl-N-nitrosourea, Dimethylnitrosamine, Dimethyl Sulphate and Methylmethanesulphonate. *Biochem. J.*, **110**, 39.

WARWICK, G. P. (1967) Covalent Binding of Metabolites of Tritiated 2-methyl-4-dimethylaminoazobenzene to Rat Liver Nucleic Acids and Proteins, and the Carcinogenicity of the Unlabelled Compound in Partially Hepatectomised Rats. *Eur. J. Cancer*, **3**, 227.

WILLIAMS, G. M., ELLIOTT, J. M. & WEISBURGER, J. H. (1973) Carcinoma after Malignant Conversion *in vitro* of Epithelial-like Cells from Rat Liver following Exposure to Chemical Carcinogens. *Cancer Res.*, **33**, 606.